# Disrupting antimicrobial resistance in food systems from a One Health perspective: a complex systems analysis in Canada, Senegal and the Netherlands

**DOI:** 10.1186/s44263-026-00322-6

**Published:** 2026-09-29

**Authors:** Chloe Clifford Astbury, Catherine Hu, Mouhamadou Moustapha Sow, Mamadou Ciss, Michel Dückers, Caroline Schneeberger, Lotte van Heuvel, Cécile Aenishaenslin, Ria Benko, Marion Bordier, Valentina De Leon, Olivier Kambere Kavulikirwa, Éva Loboda, Arabi Rajan, Arne Ruckert, Sharon Villanueva, Mary Wiktorowicz, Tarra L. Penney

**Affiliations:** 1 Global Food & Health Systems Research, School of Global Health, York University, 4700 Keele Street, Toronto, ON M3J 1P3 Canada; 2 Dahdaleh Institute for Global Health Research, York University, 4700 Keele Street, Toronto, ON M3J 1P3 Canada; 3 Global Strategy Lab, York University, 4700 Keele Street, Toronto, ON M3J 1P3 Canada; 4 National Laboratory for Livestock and Veterinary Research, Senegalese Institute of Agricultural Research, Hann Bel Air, Route des Hydrocarbures, Dakar, 3120 BP Senegal; 5 Department of Public Health and Social Medicine, Faculty of Health Sciences, Gaston Berger University, Boîte Postale 234, Saint-Louis, 32000 Senegal; 6 Netherlands Institute for Health Services Research (NIVEL), Postbus 1568, Utrecht, 3500 BN The Netherlands; 7 Département de pathologie et microbiologie, Faculté de médecine vétérinaire, Université de Montréal, 3200 rue Sicotte, Saint-Hyacinthe, J2S 2M2 Québec Canada; 8 Groupe de recherche en épidémiologie des zoonoses et santé publique, Faculté de médecine vétérinaire, Université de Montréal, 3190 rue Sicotte, Saint-Hyacinthe, Québec J2S 2M1 Canada; 9 Centre de recherche en santé publique de l’Université de Montréal et CIUSSS du Centre-Sud-de-l’Île-de-Montréal, Université de Montréal, 7101, avenue du Parc, Montréal, Québec H3N 1X9 Canada; 10 Institute of Clinical Pharmacy, Faculty of Pharmacy, University of Szeged, Szikra utca 8, Szeged, 6725 Hungary; 11 Institute of Clinical Pharmacy, Albert Szent-Györgyi Health Centre, Faculty of Pharmacy, University of Szeged, Szikra utca 8, Szeged, 6725 Hungary; 12 Emergency Care Department, Albert Szent-Györgi Health Centre, University of Szeged, Semmelweis u. 6, Szeged, 6725 Hungary; 13 Animals, Health, Territories, Risks, and Ecosystems (ASTRE), University of Montpellier, BIOS UMR ASTRE, Campus international de Baillarguet, TA A-117/E, Montpellier cedex 5, 34398 France; 14 Centre de coopération internationale en recherche agronomique pour le développement (CIRAD), ASTRE, 37 avenue Jean XXIII, BP 6189, Dakar-Etoile, Senegal; 15 Department of Social and Preventive Medicine, University of Montreal School of Public Health, succursale Centre-ville, Montréal, Québec C.P. 6128, H3C 3J7 Canada; 16 Faculty of Veterinary Medicine, Catholic University of Graben, Avenue Lubero n. 4 / Boulevard Mgr Emmanuel Kataliko, Butembo, Democratic Republic of Congo; 17 College of Public Health, University of the Philippines Manila, 625 Pedro Gil Street, Ermita, 1000 Manila Philippines; 18 WHO Collaborating Centre for Disease Prevention Policy, Systems Science & One Health, School of Global Health, York University, 4700 Keele Street, Toronto, ON M3J 1P3 Canada

**Keywords:** Antimicrobial resistance, Food systems, One Health, Systems thinking, Group model building

## Abstract

**Background:**

Food systems are a key contributor to antimicrobial resistance (AMR). Given the multi-sectoral nature of AMR, a One Health perspective has been advocated. Recent research situates AMR as an emerging property of a complex system, a perspective which can help identify root drivers of AMR and theorise the impacts of proposed solutions. This study aims to combine systems modelling and a One Health perspective to identify system-level dynamics and priority actions for addressing AMR in food systems.

**Methods:**

We used group model building, a participatory approach to systems modelling, in three countries: Canada, Senegal, and the Netherlands. Stakeholders from policy, research, civil society and industry developed qualitative systems models and identified priority actions for AMR mitigation. Workshop outputs were synthesised in the form of causal loop diagrams and iteratively refined with stakeholders to ensure they appropriately reflected their perspectives. Diagrams were analysed using systems archetypes, a conceptual tool for diagnosing recurring dynamics in complex systems, to identify common structural drivers of AMR and potential intervention points across countries. We then drew on systems theory and the priority actions identified by stakeholders to highlight potential paths to disrupting these archetypes, thereby reducing the contribution of food systems to AMR.

**Results:**

Across the countries, 39 stakeholders participated in workshops. The archetypes analysis highlighted how short-term exploitation of shared resources, reliance on antimicrobials at the expense of investing in alternative solutions, and dominance of antimicrobial-intensive production systems may contribute to AMR. Priority actions that were positioned to disrupt these archetypes included regulation, education, consumer engagement, and shift towards different production systems.

**Conclusions:**

AMR in food systems arises from recurring structural dynamics that require context-appropriate, systems-informed solutions. Countering short-term exploitation of shared antimicrobial resources calls for regulation, surveillance and education. Addressing overreliance on antimicrobials requires balanced strategies pairing judicious use with sustained investment in alternatives such as livestock management, infection prevention and vaccination. Disrupting the dominance of antimicrobial-intensive production systems calls for regulatory measures and consumer pressure to level the playing field with lower-use systems. Together, these findings point to solutions that employ a systems perspective on design, implementation and evaluation to enable long-term effectiveness in AMR mitigation.

## Background

Food systems are a key contributor to the global problem of antimicrobial resistance (AMR) [1–4]. In many countries, antimicrobial use (AMU) is common in terrestrial and aquatic livestock production [5], and antimicrobials are also finding uses in crop production [6]. Given the potential impacts of AMR on the health of humans, animals and the environment [7] a One Health approach can be an effective way to promote multi-sectoral action for tackling AMR [8, 9]. In addition, the variations in food systems across different country contexts mean that interventions to mitigate AMR in these systems must be context-appropriate, considering different resource availabilities, needs and priorities across countries [10].

While food systems play a significant role in the emergence and spread of AMR, the factors and relationships driving this phenomenon are complex. Recent research has framed the emergence and spread of AMR as an outcome of a complex system involving multiple interacting elements and actors, for example, AMR in livestock has been linked to increasing potential transmission to livestock workers and consumers [1, 2, 4, 11–13]. The behaviour of complex systems is challenging to predict given characteristics like feedback loops, delays, non-linear relationships, system adaptation, and porous boundaries [14, 15]. As a result, intervening effectively in complex systems is difficult, and can benefit from a thorough understanding of the system and its structure. A systems perspective may be valuable for understanding the drivers of AMR and informing action.

Systems archetypes are a conceptual tool that have been used to understand and communicate why complex adaptive systems exhibit recurring behaviours, which can often be problematic and challenging to shift [14, 16, 17]. By identifying archetypal behaviour in real-world systems, we can draw on systems theory to shed light on how to disrupt these problematic behaviours when they emerge [14, 16].

The aims of this study are:

1. To use systems archetypes to understand and illustrate how AMR emerges from specific paradigms, dynamics and structures within food systems; and.
2. To highlight potential paths to disrupting these archetypes, drawing on systems theory and stakeholder perspectives on priority actions for AMR mitigation in different country contexts.

## Methods

This study was undertaken as part of a multi-country project focused on strengthening AM stewardship, and draws on stakeholder insights from Canada, Senegal and the Netherlands [18]. The analysis for this study used group model building (GMB), a participatory modelling approach informed by system dynamics principles [19, 20]. The GMB processes enabled the development of causal loop diagrams (CLDs), a qualitative form of system mapping following standard notation [21], for each country. Standard notation for CLDs is described in Table 1. We then applied systems archetypes [14, 16] as an analysis tool to understand root drivers of AMR in the countries’ food systems. Our methodology was informed by a One Health perspective, which guided participant recruitment and workshop development. An author reflexivity statement was also developed to reflect on this analysis as a collaborative cross-country effort (Supplementary Material: Table S1).

**Table 1:** Standard notation for causal loop diagrams

Name	Visualisation	Description
Relationship with positive polarity		An increase in variable A causes an increase in Variable B. A decrease in Variable A causes a decrease in Variable B.
Relationship with negative polarity		An increase in variable A causes a decrease in Variable B. A decrease in Variable A causes an increase in Variable B.
Reinforcing feedback loop		Two relationships with the same polarity (in this case, two relationships with positive polarity) create a closed circle of cause and effect. This can lead to either increasing growth or increasing decline in these variables.
Balancing feedback loop		Two relationships with different polarities (i.e., one relationship with positive polarity and one relationship with negative polarity) create a closed circle of cause and effect. This leads to a balancing effect, where the feedback between variables leads to stabilizing behaviour.

### Study countries and context

Canada, Senegal, and the Netherlands were selected for this study due to the varying AMR dynamics and regulatory environments between the countries. Data was also collected in Hungary [22], but was not included in this analysis due to a lack of adequate sectoral representation. Each country has implemented policy and governance measures related to AMR and has distinct patterns of interaction between humans, animals and the environment. These are situated within different social, economic and ecological contexts.

Canada is a high-income economy with sea borders and a land border with the United States, its most significant trading partner [23]. Canada has a federal system of government, with provinces and territories having substantial discretion over some policy areas [24]. Canada is ranked 9th lowest of 204 countries for age-standardized mortality rate associated with AMR [25]. In 2023, Canada developed a national action plan (NAP) for AMR. The NAP uses a One Health approach and focuses on five pillars: research and innovation; surveillance; stewardship; infection prevention and control (IPC); and leadership [26]. Canada’s food system is classified by the Food Systems Dashboard as ‘Industrialized and Consolidated’, characterised by high levels of urbanisation; high importance of supermarkets as a food source; relatively diversified diets; and agricultural production systems with a strong reliance on technology and inputs for productivity [27, 28].

Senegal is a lower-middle-income economy sharing borders with numerous other nations in West Africa, and a member of the West African Economic and Monetary Union. As of 2019, Senegal has the 43rd highest age-standardized mortality rate associated with AMR [29]. Senegal developed its NAP to address AMR in 2017, earlier than many other countries globally. While highly focused on AMR in humans, it uses a One Health approach incorporating animal and human health considerations, including disease monitoring, strengthening laboratory capacities, surveillance of resistant pathogens, IPC, rational AMU, and raising population-level awareness and knowledge [30]. Senegal is now in the process of developing a second NAP. Senegal’s food system is classified as ‘Informal and Expanding’, indicating moderate levels of urbanisation; much lower reliance on supermarkets, with consumers purchasing foods from other sources such as markets and stalls; diets characterised by a higher reliance on staple crops; and agricultural systems with a greater reliance on workforce for productivity [27, 28].

The Netherlands is a high-income economy located in Europe and a member of the European Union (EU). The Netherlands had the 12th lowest age-standardized mortality rate associated with AMR as of 2019 [31]. The Netherlands developed its first NAP in 2015 [32], and has now published a second NAP for 2024–2030 [33]. Both NAPs were based on a One Health approach, and the current NAP aims to support innovation, IPC, judicious AMU, awareness-raising, cooperation and global collaboration. Its considerations of food systems include antimicrobial stewardship in animals, food safety and environmental transmission. Similar to Canada, the Netherlands’ food system is characterised as ‘Industrialised and Consolidated’ [27, 28], however it is situated within a different regulatory and market context, particularly given the influence of EU regulation.

### Data collection

We conducted a GMB workshop in each of the countries. Workshop format (online vs. in-person) was selected by research teams in each country. This decision and other country-specific considerations led to the development of workshop materials and processes adapted to suit each team’s context and needs (Supplementary Material 1: Table S2). Participants were identified through desktop research and professional and policy networks, recruited by email or phone, and targeted for their work related to AMR and food systems within policy, research, civil society, and industry organisations. All participants worked or lived in the case study country in question. In line with the One Health approach to this study, participants were purposively recruited to provide coverage across the human, animal and ecosystem health sectors.

This study was approved by the Human Participants Review Sub-Committee of York University’s Ethics Review Board (e2022-336) in Canada and the Comité National d’Éthique pour la Recherche en Santé (Nº 0000019/MSAS/CNERS/SP, 20 janvier 2023) in Senegal. The need for official review in the Netherlands was waived by the Medical Ethics Review Committee of Vrije Universiteit Amsterdam Medical Center. All participants provided informed written consent before participation, including consent for audio and video recordings where applicable (i.e., for online workshops).

Participant recruitment occurred on 09/10/2023 in Senegal (via official institutional letter, following previous engagement of institutions in the project); 08/11/2023-05/12/2023 in Canada (via email and phone call); and 02/05/2024-12/06/2024 in the Netherlands (via email). Workshop activities were based on published ‘scripts’ outlining best practice for GMB activities [34, 35]. These scripts can be combined to serve workshop aims and stakeholder needs and expectations. The scripts used in the workshops in this study, and their respective purposes, are described in Table 2. Full scripts can be accessed online from the open-source Scriptapedia resource (https://en.wikibooks.org/wiki/Scriptapedia).

**Table 2:** ‘Scripts’ used in GMB workshops in this study [34, 35]

Script	Purpose
Graphs over time	Engage participants in GMB by framing AMR as a dynamic behaviour over time (known as the reference mode), which serves as a starting point for discussing the causal structure underlying the behaviour of AMR over time in each country
Variable elicitation	Facilitate consensus-based discussion around which variables are important for AMR in each country, including discussion of system boundaries
Connection circle	Illustrate important variables and connections that contribute to AMR at the start of a session in a way that is easy to engage with
Causal mapping	Integrate variables and connections to develop a causal loop diagram, referring to the reference modes to reflect on drivers and feedback loops
Action ideas	Identify and prioritise actions to mitigate AMR once the model has been developed

For online workshops, a ‘seed structure’ was developed as a workshop input, an approach described in accounts of GMB practice [36] and reported in published examples [37, 38]. A seed structure is an illustration of a core set of variables and relationships that can be built on and amended through participant input and feedback, typically becoming larger and more complex through the workshop process. Seed structures can be introduced at the beginning of a GMB workshop to quickly illustrate how a problem could be represented using CLD notation.

In online workshops, we introduced the seed structure after the graphs over time exercise, and invited participants to change and build on the seed structure using the activities described in Table 2. The seed structure used in this study (Supplementary Material 1: Fig. S1) was developed based on a systematic umbrella review of peer-reviewed literature [4, 39].

For in-person workshops, data collected were pen and paper outputs developed by participants and written notes to document the conversation and process of developing these outputs. For online workshops, data collected were connection circles and CLDs developed on Kumu [40] based on participants’ input, priority actions added directly to an online whiteboard (Google Jamboard [41]) by participants, and audio-visual recording of the meeting, recorded using Zoom [42].

### Data analysis

Data entry of workshop outputs (including pen and paper and digital maps, as well as notes and recordings from the discussion) onto an online mapping platform, Kumu [40], was performed shortly after each workshop. For online workshops, preliminary data entry and analysis occurred between the first and second sessions to allow participants to review an initial iteration of the outputs and make changes and additions as needed. Where workshop participants were separated into several small groups within the workshop, all inputs were integrated into a single map to represent the workshop results.

Once initial data entry was complete for the CLDs, researchers (CCA, and CH or MMS) reviewed the mapping on Kumu, discussing and modifying as necessary to ensure that elements and relationships accurately reflected the workshop discussion and that system elements were conceptually clear and distinct. Through this process, one CLD was developed for each country to represent the elements and connections driving the emergence and spread of AMR. In Senegal, workshop outputs were translated from French (the language in which the workshop was held) by CCA, while workshops in Canada and the Netherlands were held in English.

Action ideas generated by participants were also noted in list form for each workshop, along with discussions around implementation and impact. A descriptive results report was shared with participants in each country to summarise workshop results, and participants were invited to provide any final input or feedback on the discussion.

Based on these reports, we used systems archetypes to understand and illustrate key dynamics driving AMR within food systems. Using the eight systems archetypes outlined by Kim [16] as a starting point, we inductively identified three archetypes that seemed most relevant to articulating the dynamics described by workshop participants. These archetypes were identified by CCA and CH based on their review of the data, and reviewed and discussed by the author team as a whole. We also discussed contextual similarities and differences around how these archetypes operated in each context, drawing on workshop discussions. Then, we reviewed the priority actions identified by participants. Drawing on systems theory, which posits a range of mechanisms to disrupt each archetype [14], we identified which priority actions were well-positioned to disrupt each of these archetypes, describing similarities and differences across countries in terms of actions proposed and considerations for implementation and impact.

**Fig. 1:**
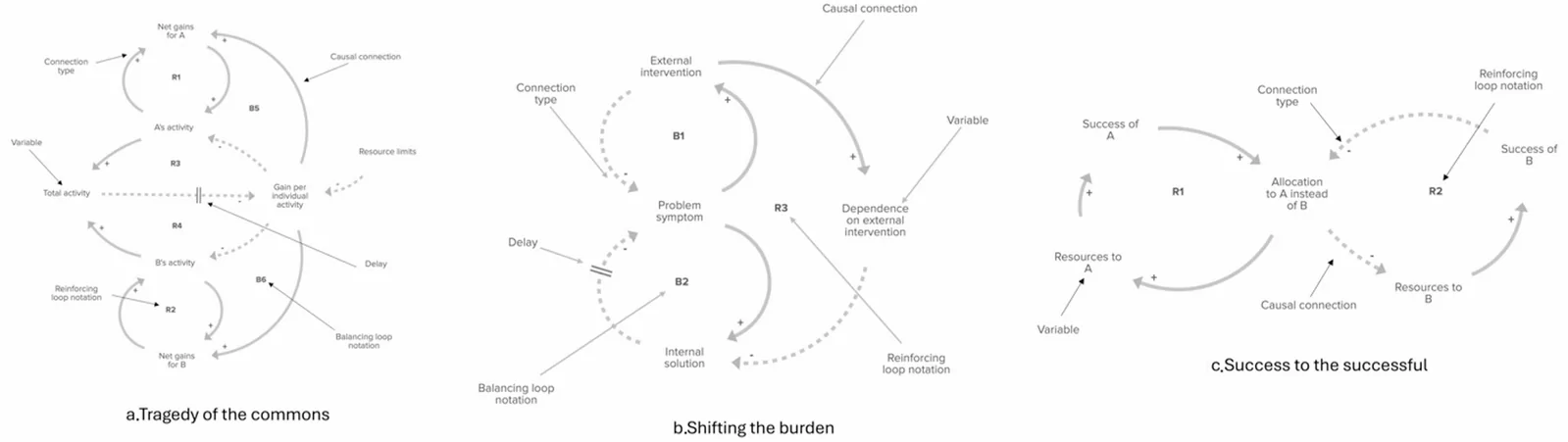
Systems archetypes applied in this analysis, drawing on Kim’s overview of common archetypes in complex systems [16]. Solid lines indicate relationships of a positive type (+), dotted lines indicate relationships of a negative type (−). Feedback loops, where factors are linked by relationships, are indicated as reinforcing (R) or balancing (B). A double bar on a relationship line indicates a delay

Systems archetypes can be represented as a series of variables and connections which combine to create a specific composition of feedback loops [16]. The three systems archetypes used here – tragedy of the commons, shifting the burden, and success to the successful – and their feedback loop structures are introduced below (Fig. 1).

### Archetype 1: tragedy of the commons

The ‘tragedy of the commons’ archetype, named after the dynamic proposed by Hardin [43], may occur where there is growth in the exploitation of a shared, but finite, resource [14, 16]. The tragedy of the commons is characterised by bounded rationality on the part of the actors involved, where individuals or organisations increase exploitation of a common good in order to maximise their own returns, without considering that, in the longer term, the depletion of that good may have irrecoverable consequences for themselves and the collective [14].

This archetype contains six feedback loops (Fig. 1a):

- Two reinforcing loops (R1 and R2) where the activity of specific actors (A and B) drives their individual net gains, which in turn incentivise them to increase their activity;
- Two reinforcing loops (R3 and R4) where the activity of these same actors (A and B) increases the total amount of activity, which in turn reduces the gain per total activity, incentivising actors to increase their activity levels, thereby further augmenting the total level of activity; and.
- Two balancing loops (B5 and B6) where the limits of a finite resource bound the potential gains for actors, no matter how much activity they engage in, which in turn leads to a decline in the net gains for A and B, disincentivising individual activity, and reducing activity overall.

While these balancing loops often exist (actors will not continue to try to exploit a resource for zero profit), this archetype becomes a tragedy when a resource has already been exploited beyond recovery before the balancing dynamic emerges, given the delay between increasing total activity and decreasing gains per individual activity.

### Archetype 2: shifting the burden

The ‘shifting the burden’ archetype reflects a dynamic in which a problem may be ‘solved’ by an external intervention which only addresses the symptom of the problem rather than its root causes [14, 16]. This diverts attention and investment away from finding internal solutions which may address the problem more sustainably, and creates ongoing reliance on the external intervention.

This archetype contains three feedback loops (Fig. 1b): a first balancing feedback loop (B1) where a problem symptom drives the need for the external intervention, which in turn reduces the problem symptom; a second balancing feedback loop (B2) where the problem symptom drives a need for internal solutions, which also reduce the problem symptom but only after a delay, as internal solutions may require greater investment and transformation and may act more slowly; and a reinforcing feedback loop (R3), where increasing reliance on the external intervention reduces attention spent on developing and implementing internal solutions, meaning these solutions have minimal opportunity to take effect.

### Archetype 3: success to the successful

The ‘success to the successful’ archetype illustrates the way in which the initial success of one actor provides benefits (e.g., resources, reputation, positive outcomes) which support sustained and escalating success [14, 16]. This can come at the expense of other actors, for whom a lack of success gets compounded over time.

This archetype contains two feedback loops (Fig. 1c): a first reinforcing loop (R1), where the initial success of one actor (A) leads to escalating success; and a second reinforcing loop (R2), where the initial lack of success of another actor (B) leads to sustained failure.

## Results

We conducted three workshops with 39 participants: 10 from Canada, 22 from Senegal, and 7 from Netherlands (Supplementary Material 1: Table S3). Participants had roles in research, civil society, policy, industry and professional bodies, and worked within and across the human, animal and ecosystem health sectors. Areas of cross-sector work included food safety at the animal-human interface.

The CLDs from each country overlapped, with similar drivers including AMU in crops and food-producing animals; the risk of transmission to human populations and increased disease burden and healthcare costs; accelerated emergence and spread in the environment; and the importance of policy drivers including surveillance, regulation and public and political will. However, certain areas of the map were substantially different across countries. For example, research and development for antimicrobials and the role of production intensification in AMR were described in Canada and the Netherlands, but not in Senegal. Meanwhile, unregulated pharmaceuticals and medical practice were a concern for workshop participants in Senegal. Participants generated a list of 63 actions (Supplementary Material 1: Table S4).

Based on these workshop outputs, we identified the three systems archetypes that seemed most relevant to describe key dynamics outlined by workshop participants. We then reviewed the list of actions proposed by participants and identified the actions likely to be most effective based on systems theory. See Supplementary Material 1: Table S5 for worked examples of this process.

### Archetype 1: tragedy of the commons

**Fig. 2:**
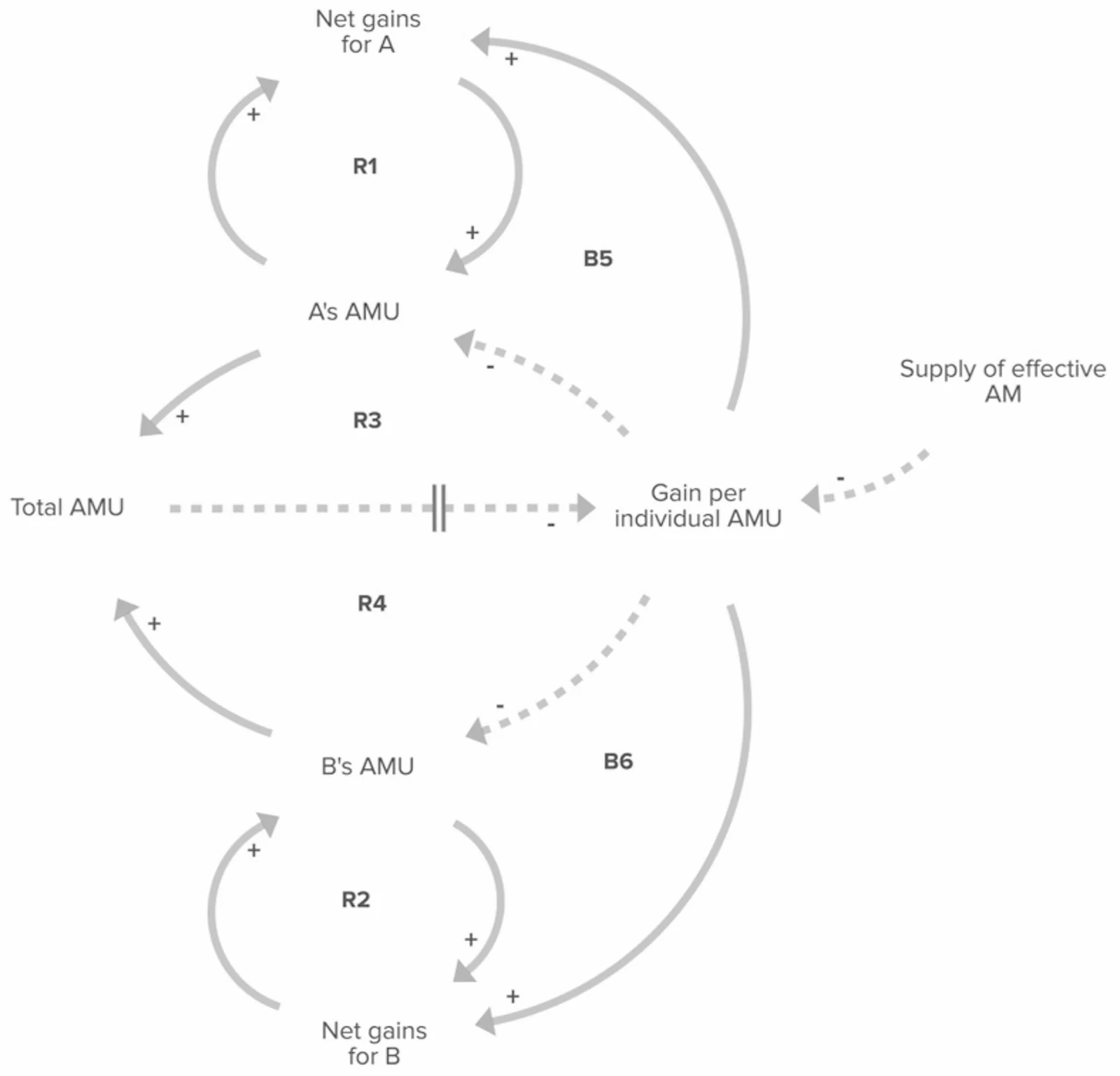
Application of the tragedy of the commons archetype to the dynamics of AMR within the food system. Solid lines indicate relationships of a positive type (+), dotted lines indicate relationships of a negative type (−). Feedback loops, where factors are linked by relationships, are indicated as reinforcing (R) or balancing (B). A double bar on a relationship line indicates a delay

AMU can be understood as an example of the tragedy of the commons archetype (Fig. 2): individual actors may gain substantially from AMU (R1 and R2), with advantages including reduced morbidity and mortality, with ripple effects for increased productivity and profitability when used in livestock and crops. However, AMU is known to be a key driver of AMR, as pathogens exposed to antimicrobials can develop resistance. Over time, as resistance emerges and spreads, the supply of effective antimicrobials may decline, and actors may turn to second- or third-line treatments to maintain antimicrobial effectiveness (R3 and R4). This will eventually reduce the individual gain from AMU, as resistant pathogens increasingly require longer and more complex treatment regimens, resulting in productivity decrease (B5 and B6). However, because of the delay before these impacts are felt, the supply of effective antimicrobials may be substantially reduced before these balancing dynamics emerge.

Participants in all countries recognised the incentives driving individual food producers to use antimicrobials for disease treatment and prevention. AMU was also used for growth promotion in Senegal, as well as in Canada, though to a lesser extent, as growth promotion claims have been removed from antimicrobial labels and extra-label usage is prohibited. Participants in the Netherlands thought AMU for growth promotion was almost non-existent in the Dutch context, given that this practice is banned under EU regulation, making any use illegal.

Beyond individual users, the role of countries as actors in this archetype was raised: the gains experienced by countries from AMU could take the form of being able to produce and sell products at a lower cost, making them more competitive exporters in a global market. In this way, the regulatory environment in different countries could threaten a global supply of effective antimicrobials. Indeed, the threat of injudicious AMU in other countries and the possibility of AMR spreading from other jurisdictions was discussed in all workshops. Across the three countries, participants discussed the role of globalization in increasing the spread of AMR through cross-border travel and trade of livestock and food products.

This dynamic may also harm food security. Participants discussed impacts of AMR including reduced production outputs and increased production costs. These factors could increase food costs, causing a growth in food insecurity. Food safety may also be impacted through the transmission of pathogens to humans through the food chain.

A tragedy of the commons archetype can be addressed in several ways [14, 16], which can be used to identify actions proposed by stakeholders (Supplementary Material 1: Table S4) that may disrupt this dynamic. First, educate and exhort: individual actors can be educated or entreated to change their behaviour. In all countries, education was a widely discussed priority action for AMR. Participants discussed how educational initiatives should be suitable for different audiences, including schoolchildren, the general public, and a range of professionals. This included mass media campaigns, which were seen as particularly important given the rise of misinformation and social media use in all countries. However, in Senegal some participants questioned the effectiveness of educational interventions, given that many such interventions had been delivered with minimal impact for behaviour. In Canada, participants raised the importance of incorporating behavioural science techniques into the development of educational interventions to improve effectiveness.

Second, regulate the commons: regulations such as bans, quotas, disincentives or incentives can be used to change the practices of individual actors. This may also include supra-national regulation and governance, which were discussed, for example, in the Netherlands, an EU member state. This option relies on regulators being knowledgeable about the state of the commons, and having the capacity and integrity to enforce regulations [14]. The importance of regulators understanding the state of AMR was underscored in all countries, with participants emphasising the importance of surveillance systems for AMR, AMU and antimicrobial residues across sectors. In Canada and the Netherlands, the need to strengthen capacity for diagnostic testing at point of care for humans and animals was discussed as part of this, supporting more judicious AMU. However, as well as requiring a sound knowledge of the state of the commons, the capacity to enforce regulations is also important to disrupt the tragedy of the commons through regulation [14]. In the Netherlands, for example, food exports were recognised as an important contributor to the national economy, which gave the food animal production sector power in shaping regulation. In Senegal, the existence of a black market in antibiotics was acknowledged, which could be challenging to regulate as these illicit products were often imported across land borders. These antibiotics were sold without prescriptions and were often of lower product quality.

### Archetype 2: shifting the burden

**Fig. 3:**
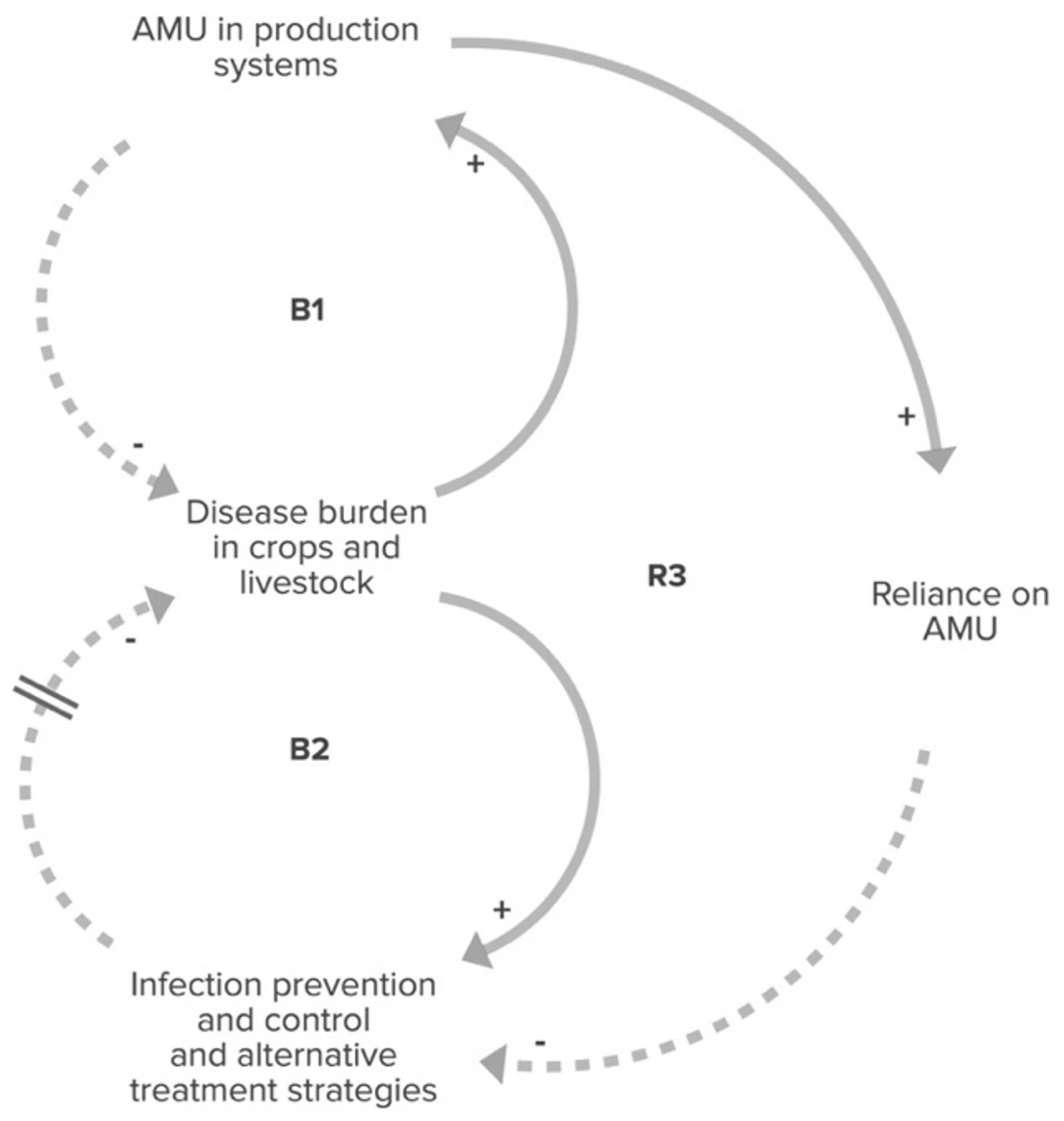
Application of the shifting the burden archetype to the dynamics of AMR within the food system. Solid lines indicate relationships of a positive type (+), dotted lines indicate relationships of a negative type (−). Feedback loops, where factors are linked by relationships, are indicated as reinforcing (R) or balancing (B). A double bar on a relationship line indicates a delay

The reliance on AMU to prevent and treat disease in crops and livestock presents an example of the ‘shifting the burden’ archetype (Fig. 3): the burden of disease in crops and livestock demands a solution, and AMU provides an effective and relatively rapid mode of treatment (B1). However, subsequent use has resulted in overreliance and downstream emergence of AMR. While other modes of treatment and prevention exist, these may take longer to take effect, or be used as preventive strategies rather than treatments (B2). Production systems come increasingly to rely on AMU to keep crops and animals healthy, with declining investment in other strategies (R3).

This archetype can be disrupted by strengthening the system’s ability to address its own problems, without undue reliance on the external intervention. Study participants discussed alternative methods for disease prevention and treatment extensively in all workshops. Many specified that the aim was not for food systems without AMU, but rather where antimicrobials were used *judiciously*, in combination with other approaches. In all countries, participants discussed the importance of strengthened IPC or biosecurity to reduce reliance on AMU. Better IPC and waste disposal regulation was also discussed as a way to avoid the spread of AMR into the environment, including via water, soil and wildlife. Alternative approaches to disease prevention and treatment were also discussed, including vaccination, phage therapy and gene editing.

Across all countries, participants highlighted the importance of political and public will to take action on AMR, as strengthening IPC could require investment, including supporting producers to make changes to infrastructure and processes. In Senegal, the issue of resource barriers was discussed, with many areas of capacity-building and infrastructure development seen as currently requiring investment. In Canada, potential equity impacts of tighter antimicrobial regulation and focus on IPC and alternative treatment strategies were discussed, given that smaller farms may have less resources at their disposal to invest in the infrastructure change needed to move away from excessive reliance on AMU. This investment may be more feasible for large-scale farms, while smaller farms may struggle to cope with the costs.

### Archetype 3: success to the successful

**Fig. 4:**
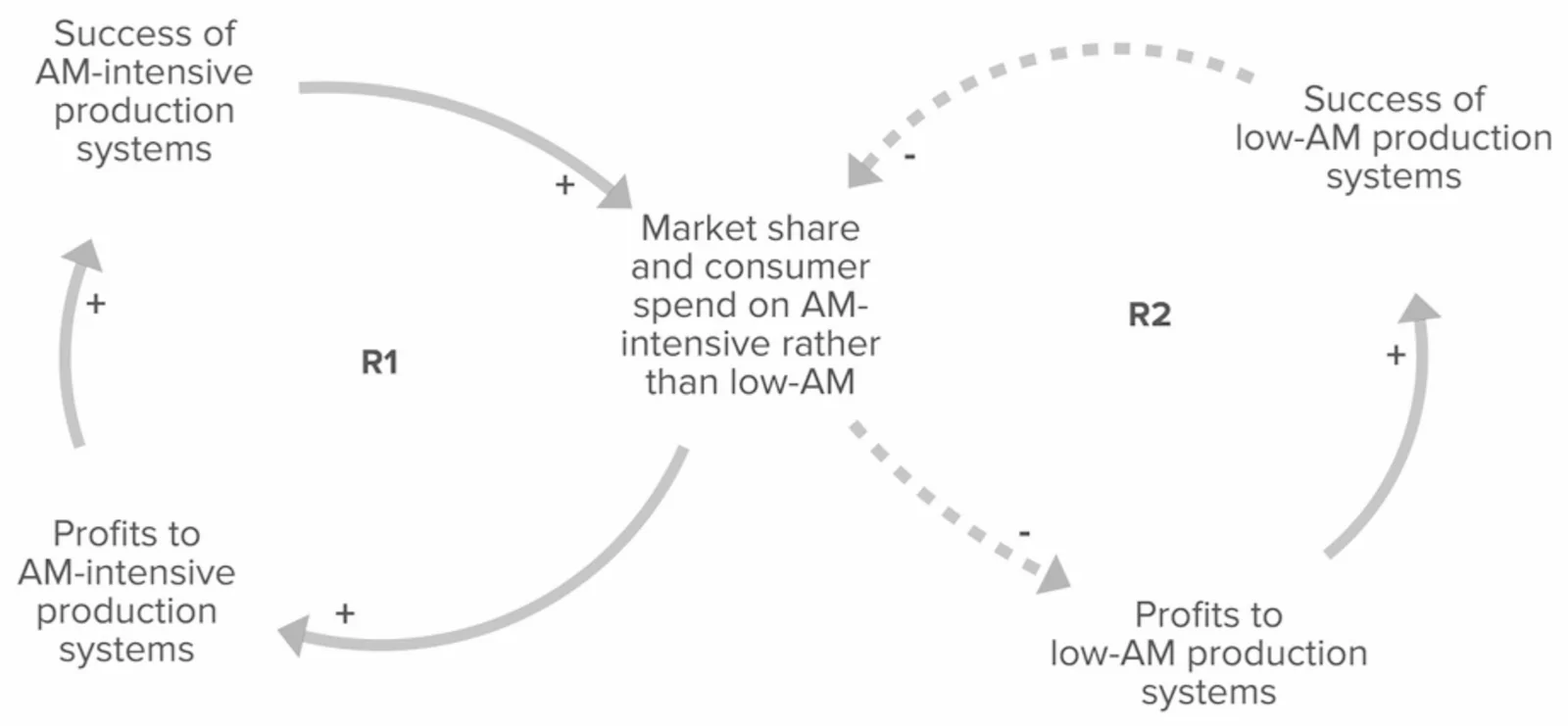
Application of the success to the successful archetype to the dynamics of AMR within the food system. Solid lines indicate relationships of a positive type (+), dotted lines indicate relationships of a negative type (−). Feedback loops, where factors are linked by relationships, are indicated as reinforcing (R) or balancing (B)

The predominance of antimicrobial-intensive production systems in food systems can be understood as a case of success to the successful (Fig. 4): as antimicrobials allows producers to reduce costs and sell their products at lower prices, antimicrobial-intensive production systems come to dominate the market and shape consumer preferences and expectations around food costs (R1). This shapes consumer spending, particularly for consumers living in food insecurity. Low-antimicrobial production systems may then struggle to remain viable (R2).

One strategy for disrupting this archetype involves levelling the playing field: removing some of the advantages of the strongest actors while strengthening the weaker actors. Regulations around withdrawal periods – periods of time after AMU where animals and their products cannot be used for human consumption – existed in all three countries and can contribute to reducing the emergence and spread of AMR in human populations. However, these regulations may also play an unintentional role in balancing competitive conditions, as antimicrobial-intensive production systems may have to wait to sell their products until the withdrawal period has elapsed, which can result in a loss of profit. This advantages production systems that rely less intensively on AMU, and may also incentivise antimicrobial-intensive production systems to reduce their AMU. In Senegal, the need to enforce existing withdrawal period regulations was discussed, with the establishment of a system to monitor the presence of antibiotic residues in food proposed, as described above, as an enforcement mechanism.

Another approach to levelling the playing field can involve re-shaping consumer demand. In the Netherlands, the possibility of transitioning to production systems with lower density, including agroecology, and slower growth livestock varieties was discussed as a mechanism to reduce reliance on AMU, but it was also recognised to have potential negative impacts for profitability. However, the Netherlands had nevertheless experienced a success story in making this transition in the poultry sector, with slower-growing varieties now dominating the domestic market. This had been achieved through engagement from civil society, with organisations pushing for a shift in production systems, and a resulting change in consumer norms and expectations, such that slow-growing varieties are now chosen by many mainstream consumers. Further improvements in animal production systems to reduce reliance on AMU remained a priority action for participants in the Netherlands, though it was seen as a long-term project. Participants also saw consumer pressure as something that could continue to be levied through labelling strategies to raise consumer awareness of practices in livestock production, for example the Better Life (Beter Leven) labelling scheme, through which producers could certify their animal products as being produced through higher-welfare practices [42].

## Discussion

Our study identifies key system dynamics that drive AMR in food systems and makes recommendations for action based on this understanding. First, the “tragedy of the commons” archetype illustrated how individual and national actors exploit shared resources (e.g., antimicrobials) for short-term gains, leading to long-term depletion of effective antimicrobials and the emergence and spread of AMR. Study participants emphasized the need for education, regulation, and surveillance to mitigate this dynamic. Second, the “shifting the burden” archetype revealed how reliance on AMU can prevent investment in more sustainable solutions, such as infection prevention and vaccination, with participants stressing the need for balanced strategies incorporating judicious AMU alongside other strategies. Finally, the “success to the successful” archetype demonstrated how AM-intensive production systems can come to dominate markets because they are more profitable than lower-AMU systems. Participants suggested regulatory measures and consumer pressure as ways to level the playing field.

Previous applications of systems theory to AMR have argued that resilience—the ability of a system to resist disruption, for better or for worse—can play a role in both driving and mitigating this problem [11, 44]. This perspective aligns with our analysis, which identifies system-level behaviours as some of the root causes of AMR. This presents an example of a negative incarnation of resilience, in which problematic dynamics are challenging to shift. Our study builds on this conceptualisation to identify interventions that have the potential to disrupt these problematic archetypal behaviours.

The recently published AMR-Intervene framework [11] represents an application of systems theory in order to identify potentially effective interventions for AMR mitigation, incorporating the systems idea of leverage points [14, 45] to classify interventions as either high or low leverage. Applications of this framework to existing interventions have found that in some contexts, the majority of interventions may be considered low leverage, having limited potential to provoke system-level change, though description of the nature of these low-leverage interventions and the leverage points they target is limited [46].

Beyond applications of systems theory, existing scholarship has conceived of AMR in ways that align with the findings of this study. For example, our use of the ‘shifting the burden’ archetype to describe how AMU enables food systems processes and practices to persist aligns with Chandler’s concept of AMs as infrastructure [47]. Meanwhile, the use of the ‘tragedy of the commons’ archetypes aligns with ongoing discourse around AMR as a problem of the global commons, requiring collaborative and aligned solutions [48, 49]. Finally, many of the interventions proposed by workshop participants align with previously identified One Health approaches to antimicrobial stewardship, including measures such as regulating use and strengthening surveillance [50].

This study provides a systems theory perspective on how to intervene to mitigate AMR, focusing on interventions proposed by country stakeholders. This perspective highlights how some interventions are positioned to disrupt root drivers, while others may only provide short-term solutions. Study participants overall agreed that AMU-free food systems were not the aim, and efforts should focus on judicious AMU. As part of this, research and development for new antimicrobials was discussed by participants, which would contribute to growing the pool of effective antimicrobials. However, interventions to support antimicrobial development would not disrupt the system-level behaviours considered in this study. While they are an important part of the effort, if the root drivers are still in place, resistance may continue to develop. This has implications for how we invest in AMR mitigation as a global community [10].

This study also underscores how a systems perspective can support intervention design and adaptation to promote sustainable impacts [51], aligning with previous arguments on the need for adaptive governance in the face of AMR [44]. Our analysis situates AMR-focused interventions as disruptions within a complex system, where different actors and elements adapt to achieve their own objectives. In food systems, commercial actors are often driven by profit, which may be at cross-purposes with the aim of antimicrobial stewardship [4]. Meanwhile, pathogens in this social-ecological system also adapt to disruptions [44]. In some cases, system adaptation may even lead to intervention failure [52]. Systems thinking has been acknowledged as a core competency for One Health research and practice [53], though it continues to be under-represented in One Health training [54]. The systems modelling approach applied here is one of a series of methods which are increasingly used in population health research [21], which could be applied more broadly to shed light on AMR and other One Health topics.

The importance of the archetypes described in this study for the issue of AMR, and the potential of the actions discussed to disrupt them, are based on insights from systems theory. Members of the community of research and practice that employ archetypes report that they are effective tools for understanding the behaviour of complex systems, and that the actions recommended provide effective solutions to the problems they describe [14, 16, 17, 55]. However, the application of systems theory to the topic of AMR is emergent. Future research can build on this study by carrying out systems-informed evaluation of the interventions discussed. Within this approach to evaluation, interventions are understood as disruptions within complex systems, and systems-informed evaluation considers how both the system and the intervention may adapt to each other over time [56, 57]. Archetypes can be useful tools within systems-informed evaluation, helping to target interventions, predict system behaviour, identify enablers and barriers, and inform evaluation design [58].

This study incorporates data from multiple countries facing different challenges and opportunities for AMR mitigation, highlighting commonalities and contextual differences in combatting this global problem. In addition, the workshops included participants from multiple organisations and sectors, providing a wide range of perspectives on this issue. However, there were differences in data collection procedures across country contexts. In particular, workshop format (in-person vs. online) varied, and online workshops were made shorter to mitigate participant fatigue in an online setting. To work effectively in this shorter time frame, we introduced the ‘seed structure’ as a starting point participants to build on, rather than starting from scratch. In addition, participant composition was different, with the Netherlands in particular lacking representation from industry and civil society, which were present in the other countries. These differences were due to the adaptation of study procedures to country-specific budgets and priorities and networks, and variation in networks and responses to recruitment efforts. This may have led to some perspectives being more emphasised in some workshops than in others.

## Conclusions

This study highlights key system dynamics driving AMR in food systems, using three systems archetypes to describe root causes of AMR in food systems, and explores the potential of interventions to disrupt these dynamics, drawing on the perspectives of stakeholders in Canada, the Netherlands and Senegal. These archetypes explain how short-term exploitation of shared resources, reliance on AMU at the expense of investing in alternative solutions, and dominance of antimicrobial-intensive production systems may contribute to the persistence and spread of AMR. Overall, the findings underscore the complexity of AMR dynamics, highlighting interventions that have the potential to disrupt these dynamics including regulatory frameworks, education, and a shift towards more sustainable agricultural practices. The findings suggest that interventions may benefit from a systems perspective on design, implementation and evaluation to enable long-term effectiveness in AMR mitigation.

## Supplementary Information

Below is the link to the electronic supplementary material.

Supplementary Material 1: Additional file 1: Table S1-S5, Figure S1.

## Data Availability

All outputs generated in workshops (priority action lists, systems maps, and reflection on workshop processes) are publicly available in workshop reports at: https://design.dighr.org/project-outputs/. Original workshop recordings and notes are not publicly available to protect the confidentiality of participants operating in relatively small policy communities in their respective countries. If there are any questions regarding the original material, please email tpenney@yorku.ca.

## Abbreviations

AMR: Antimicrobial resistance
AMU: Antimicrobial use
CLD: Causal loop diagram
GMB: Group model building
NAP: National Action Plan
EU: European Union
IPC: Infection prevention and control

## Declarations

### Authors’ information

While participant recruitment and workshop organisation were the product of close collaboration with teams from each country, the lead and senior authors (CCA & TLP) are based in Canada. Co-authors from all participating countries provided feedback on analysis and interpretation (see author reflexivity statement in Supplementary Material 1: Table S1).
