# Supplementary material for: Disrupting antimicrobial resistance in food systems from a One Health perspective: a complex systems analysis in Canada, Senegal and the Netherlands
Source: BMC Glob Public Health. 2026 Sep 29;4:94. doi: 10.1186/s44263-026-00322-6 (PMC13621477; doi:10.1186/s44263-026-00322-6)
Supplement: Supplementary file 1 [file 44263_2026_322_MOESM1_ESM.docx]

# Supplementary Material 1

## Table S1: Structured author reflexivity statement

| Study conceptualisation | 1. **How does this study address local research and policy priorities?**   This study aims to understand key drivers of AM resistance situated in food systems and strengthen the design and evaluation of AM stewardship policies from this perspective. This is an issue of key importance in the three countries where data collection was undertaken (Canada, the Netherlands and Senegal), and all countries are currently engaged in policy efforts to mitigate AMR. |
| --- | --- |
|  | 2. **How were local researchers involved in study design?**   The lead and senior researchers (CCA and TLP) discussed study design at the outset of the project with local researchers in each of the countries involved and developed workshop processes and materials based on these discussions. For this reason, workshop procedures were slightly different across countries (see Supplementary File 2) to ensure that the research needs of country teams were being met. |
| Research management | 1. **How has funding been used to support the local research team(s)?**   This project was funded by the Joint Programming Initiative on Antimicrobial Resistance (JPIAMR), through which research teams in each country participating in the project were directly funded to support hiring and other research costs. |
| Data acquisition and analysis | 1. **How are research staff who conducted data collection acknowledged?**   All research staff who conducted data collection (i.e., workshop planning, workshop facilitation and data entry) in all three countries are included as authors. |
|  | 2. **How have members of the research partnership been provided with access to study data?**   The project has a data ownership and access policy to provide a transparent process for data to be shared within the research team. Analyses of study data led by other members of the research partnership are currently in progress. |
|  | 3. **How were data used to develop analytical skills within the partnership?**   All members of the research consortium, including research teams from Canada, the Netherlands, Senegal, Hungary, the Philippines and France, were offered an online session introducing systems analysis and group model building procedures for those who were not familiar with this approach or interested in learning more. All country teams were offered the options of receiving facilitation training in order to run the workshops themselves, or have the York research team facilitate the workshop if that was their preference. In Senegal, the ISRA team opted to receive training and facilitate the workshop. The lead author (CCA) ran a two-day training session in French in Dakar with all members of the Senegalese facilitation team. Other colleagues based in Dakar were also invited if they were interested in learning more about the methods. The ISRA team then facilitated the workshop (see Supplementary File 2) with on-site support from CCA where needed. In Canada and the Netherlands, virtual workshops were facilitated by the lead author (CCA) and early-career researchers based at York University (CH, VDL, AR) with support from country collaborators (CS, LVH). |
| Data interpretation | 1. **How have research partners collaborated in interpreting study data?**   The analysis of the data and the interpretation using the systems archetypes was led by the lead and senior researcher and their colleagues at York University in Toronto, Canada. Before sharing the draft manuscript with the co-author group as a whole, a report summarising the workshop in each country in a standardised way to support the comparative analysis was shared with the relevant country team for review and input. In Senegal, this report built on existing data entry and analysis led by MMS to summarise the workshop for participants and analyse the data for a Senegal-specific analysis (manuscript in preparation). These reports fed into the analysis presented in this manuscript. The manuscript was also shared with the author team as a whole, including research partners who had been involved in workshop planning and facilitation in all countries included in the project, for input and feedback. |
| Drafting and revising for intellectual content | 1. **How were research partners supported to develop writing skills?**   This manuscript was mainly drafted by the lead author (CCA), with contributions from the second author (CH), both early-career researchers based at York University. All authors, including those in the case study countries, read the draft and provided critical input on style and content, thereby supporting writing development for the early-career researchers involved as authors. |
|  | 2. **How will research products be shared to address local needs?**   This analysis will be published in an open access format. Evidence from this analysis will be integrated with broader project findings to develop policy briefs and an informative poster that will be made publicly available. |
| Authorship | 1. **How is the leadership, contribution and ownership of this work by LMIC researchers recognised within the authorship?**   Researchers from all countries that participated in the project (Canada, the Netherlands, Senegal, Hungary, the Philippines and France) are included in the author list, reflecting their substantial contributions to study design, participant recruitment and data collection. The project has a publication policy based on ICMJE criteria, whereby all project members may express interest in any planned publication incorporating data from multiple countries, and be part of the author team providing they have made author-level contributions based on these criteria. The author list is structured to acknowledge the first two authors as having carried out coding and analysis for this comparative study (CCA, CH) as well as having a substantial role in workshop development, coordination and facilitation, followed by a third author who led data entry and analysis for the Senegalese case study (MMS), followed by other country team members involved in data collection (including workshop coordination, participant recruitment and facilitation) in alphabetical order, followed by other project members who reviewed the manuscript and contributed critical input in alphabetical order. However, we acknowledge that the lead and senior authors (CCA and TLP) are both based at an high-income country institution, reflecting their contributions to the design, coordination and analysis of this comparative study. |
|  | 2. **How have early career researchers across the partnership been included within the authorship team?**   The first and second authors (CCA and CH) are both early career researchers (post- and pre-doctoral respectively) and their contributions to this work are reflected in their authorship positions. Other ECRs who contributed to data collection and coordination in the case study countries (MMS, LVH, EL) and contributed to analysis within the York team (VDL, AR) are also included within the authorship team. |
|  | 3. **How has gender balance been addressed within the authorship?**   The authorship team is composed of 12 women (including both the lead and senior authors) and 5 men, working in both HICs and LMICs. |
| Training | 1. **How has the project contributed to training of LMIC researchers?**   The authorship team is composed of junior and senior researchers based in both HICs and LMICs. Project funds were used to hire early-career researchers (postdoctoral fellows, postgraduate and graduate students, and research assistants) in all countries, providing hands-on training in research activities including applying for ethics, participant recruitment, data collection, data management and project management. The lead author (CCA) ran a two-day training workshop in Dakar prior to the Senegalese workshop, including introducing the GMB method and adapting workshop materials in collaboration with the team from the Institut Sénégalais de Recherches Agricoles (ISRA). The ISRA team then facilitated the workshop, contribution to data collection quality through a deeper knowledge of the context and understanding of stakeholders. This process provided both formal and hands-on training around the study method. |
| Infrastructure | 1. **How has the project contributed to improvements in local infrastructure?**   The project has not contributed to improvements in local infrastructure, beyond the capacity-strengthening through training described above. |
| Governance | 1. **What safeguarding procedures were used to protect local study participants and researchers?**   As well as complying with York ethics guidelines, appropriate ethics procedures were also followed in each of the study contexts based on the guidance from the country leads in Senegal and the Netherlands. This including obtaining formal ethics approval in Senegal, although formal ethics approval for the planned data collection was waived in the Netherlands. |

## Table S2: Workshop characteristics

| **Characteristics** | **Senegal** | **Canada** | **Netherlands** |
| --- | --- | --- | --- |
| **Format** | In person | Online | Online |
| **Length** | 2 days | 2 x 2 hours | 2 x 2 hours |
| **Language** | French | English | English |
| **Coordination team** | MC, MMS | CCA, CH | LVH, CS |
| **Facilitation team** | MMS, MC, AG, RS, AIS, KB, CCA | CCA, CH, VDL | CCA, CH, VDL |
| **Data entry team** | MMS, KB, CCA | CCA, CH | CCA, CH, AR |
| **Participants targeted** | Research, civil society, policy, industry, professional association | Research, civil society, policy, industry, professional association | Research, policy |
| **Framing of workshop** | One Health and antibiotic resistance | Food systems, One Health and AMR | One Health and AMR |
| **Mapping approach** | Physical (Sticky notes, markers, paper) | Online mapping platform | Online mapping platform |
| **Data capture approach** | Physical outputs and written notes | Online maps and workshop recording | Online maps and workshop recording |
| **Use of a seed structure** | No | Yes | Yes |
| **Date** | 25-10-2023, 26-10-2023 | 20-02-2024, 22-02-2024 | 17-06-2024, 20-06-2024 |

## Seed structure

The seed structure was informed by the results of a systematic scoping review [4,39] which aimed to synthesise the evidence on AMR drivers within food systems from a complex systems perspective, highlighting the interconnections between factors that contribute to AMR emergence and spread. An umbrella review methodology was used to identify relevant studies. We searched Medline, SCOPUS, Agricola, and Dimensions using terms related to AMR, food systems, and reviews. Systematic reviews containing at least one relationship between variables in food systems were included. Data were extracted and summarised according to umbrella review guidelines, and a causal loop diagram (CLD) was developed to map the interrelationships between food systems factors and AMR.

We coded relationships evidenced in each review, focusing on the empirical findings present in each study, which could involve one or more relationships per study. We characterised each relationship as:

- Exposure – the cause/action that would result in an outcome
- Outcome – resulting from the exposure
- Relationship type (+ or -) – positive relationship indicates that the directionality of an exposure would result in the same directional outcome (a positive exposure 🡪 a positive outcome) whereas a negative relationship indicates the opposite (a positive exposure 🡪 a negative outcome)
- Excerpt evidencing the relationship

The factors (exposures and outcomes) identified during this process were categorised based on the One Health sector to which they belonged – human health, animal health, or environmental health – or as economic factors, reflecting the importance of economic consideration in food systems practice and decision-making.

## Figure S1: Seed structure

## Table S3: Participant characteristics

|  | **Canada** | **Senegal** | **Netherlands** | **Total** |
| --- | --- | --- | --- | --- |
| **Organisation** | | | | |
| **Research institution** | 1 | 6 | 3 | 10 |
| **Civil society** | 2 | 1 | 0 | 3 |
| **Policy** | 1 | 12 | 4 | 17 |
| **Industry** | 4 | 2 | 0 | 6 |
| **Professional association** | 2 | 1 | 0 | 3 |
| **Sector** | | | | |
| **Human** | 2 | 8 | 2 | 12 |
| **Animal** | 5 | 8 | 2 | 15 |
| **Environment** | 2 | 1 | 0 | 3 |
| **Cross-sector** | 1 | 5 | 3 | 9 |
| **Total** | 10 | 22 | 7 | 39 |

## Table S4: Actions proposed by stakeholders during participatory modelling workshops in each of the three countries included in the study

**Canada**

1. Quantify sources of AMR in different parts of the food system
2. Improve environmental surveillance of AMR and AM residues
3. Changes in regulatory fees and regulatory burdens to introduce new products in the Canadian market
4. Implementation of management practices in human and animal health that reduce AMU without compromising health
5. Science-based education on AMR at every level of schooling
6. Enhance and further develop educational programs/access to information regarding AM and judicious use
7. Support for the translation of Canadian research innovation in AMR to industry in Canada
8. Increasing health workforce (veterinary and human) through regulatory changes
9. Streamlining and harmonizing regulatory procedures with other regulatory agencies to enhance access to novel AM or alternatives to AM
10. Increased AMU and AMR surveillance across all sectors
11. Regularly publish AMU patterns by the various segments of both human and animal-focused users, informed by surveillance data
12. Creating market and incentives for the development and introduction of new AM and non-AM alternatives (including vaccination, gene editing and phage therapy)
13. Mass media campaigns to undo misinformation and educate population on the use of AM, and on the food systems in Canada
14. Federal strategy implemented to increase Canada's access to new AM.
15. Rapid and verified diagnostic techniques that increase prudent AM use and valuable alternative practices.
16. Research and development in diagnostic tools to lead to faster diagnostics for surveillance
17. Development of rapid diagnostic tests human/animal health
18. Informed regulatory and policy development
19. Investing into the education system to build understanding and public trust towards our food systems and how AMR is part of them
20. Broad education for different parties (public, producers, veterinarians, health care workers) on AMR and its impact with a focus on behavioural change

**Senegal**

1. Promoting sanitation in local areas
2. Raising awareness of antibiotic resistance and good practices among stakeholders
3. Monitoring and quality control of medicines
4. Communicating the risk of antibiotic resistance to the population
5. Improving waste management
6. Developing programmes to reduce poverty
7. Conducting vaccination campaigns
8. Outreach on ABR and One Health for professionals across sectors
9. Promoting the training of grassroots actors
10. Establishing a national network of professionals focused on ABR
11. Raising awareness on existing laws and regulations
12. Establishing a system to monitor the presence of antibiotic residues in food*
13. Mobilizing producer organisations in the fight against ABR
14. Strengthening health services with equipment and materials, including in laboratories
15. Providing the population with quality health facilities
16. Providing farmers with quality veterinary services
17. Strengthening working groups on hospital-based infections (Indicated in workshop outputs as “CLINs”: Comités de lutte contre les infections nosocomiales)
18. Making access to care equitable for both humans and animals
19. Strengthening data infrastructure
20. Implementing an integrated waste management system (biomedical, animal, environmental, etc.)
21. Mapping, harmonizing and revitalizing ABR regulations
22. Strengthening drug sales control strategies including enforcing sanctions against the illicit sale of medicines
23. Strengthening the capacities of stakeholders around ABR and the One Health approach
24. Enforcing withdrawal period regulations
25. Strengthening human resources in remote areas
26. Improving water quality

**The Netherlands**

1. Informed regulatory and policy development
2. Strengthen monitoring and surveillance systems for AMU and AMR including for Gram-positive bacteria
3. Facilitate the sharing of microbial sequence data at national and international levels
4. Implement and strengthen infection prevention and control measures
5. Ensure the rational use of antibiotics in the human healthcare sector
6. Develop and advance point-of-care diagnostics
7. Increase awareness and understanding of AMR
8. Enhance the speed of detecting potential resistance clusters through whole-genome sequencing
9. Address the impact of stigma related to being a carrier of multi-resistant strains, and consider related laws and privacy issues
10. Foster the development of new antibiotics
11. Enhance AMU practices in LMICs, as part of global efforts given the cross-border nature of AMR
12. Transform food systems towards regenerative agriculture
13. Increase the availability of over-the-counter diagnostics for infections
14. Establish a comprehensive animal public health system
15. Improve animal production systems to reduce reliance on AMU
16. Promote vaccination to prevent various infections
17. Investing and expanding research programs

## Table S5: Worked examples of the analytic chain linking causal loop diagrams, archetypes, and actions

| **Archetype identified** | **CLD evidence** | **Rationale for application of the archetype** | **Intervention implications** |
| --- | --- | --- | --- |
| Tragedy of the commons | CLDs showed that antimicrobial use (AMU) provides immediate benefits to individual actors (e.g., disease control, productivity, profitability), encouraging continued use: AMU to reduce the burden of disease in food systems is described in all the CLDs. Productivity and profits were also mentioned, though the specific mechanisms looked different across contexts (e.g., in Senegal, AMU for growth promotion was acknowledged, whereas in the Netherlands this was strictly regulated, but AMU could still support profits by improving health and enabling production intensification, for example).  At the same time, cumulative AMU increases antimicrobial resistance (AMR), progressively reducing the shared stock of effective antimicrobials and ultimately diminishing the benefits of AMU for all users: the link between AMU overuse and misuse and AMR was reflected in all CLDs.  Delays between AMU and the emergence of resistance obscure these long-term consequences, so in the short term individual actors continue to rely on AMU to obtain the benefits noted above. Again, this varied across contexts, with Dutch stakeholders perceiving that strict regulation had already substantially reduced AMU, while both Canadian and Senegalese stakeholders fearing that AMU and AMR would continue to increase given their current regulatory context. | This structure matches the Tragedy of the Commons archetype because multiple actors independently draw benefit from exploiting a finite shared resource (the pool of effective antimicrobials), while the cumulative effects of their actions degrade that resource for everyone (as overuse and misuse drive AMR). The delayed feedback between resource exploitation and its depletion enables overuse before balancing effects become apparent. | Systems theory suggests that this archetype can be disrupted by:  (1) educating and exhorting users to encourage stewardship of the shared resource;  (2) regulating the commons; and  (3) privatising the commons.  For (1), we searched for actions which relied on increasing awareness and understanding of the commons as a means of changing the behaviour of the , rather than relying on coercion (regulation) or changing incentives (privatisation). In this category, we identified education for diverse audiences.  For (2), we searched for actions which regulated AMU or enabled regulation. Existing regulations were mentioned (e.g., restrictions on the use in livestock of antimicrobials important for human medicine, mandatory withdrawal periods, stipulations for those working in agriculture and in waste management to wear personal protective equipment) during the modelling process. However, in discussing priority actions, participants emphasised additional key measures that would better enable regulation, namely improved surveillance systems and diagnostics, and strengthened capacity and budget to enforce existing regulations.  On reviewing actions proposed by participants, we did not identify actions that aligned with (3), which would involve privatising different parts of the antimicrobial commons to shift incentives and encourage better stewardship. This option for the disrupting the archetype is therefore not discussed in the body of the text. |
| Shifting the burden | CLDs showed that AMU provides a rapid and effective response to disease in livestock (and crops, in certain cases), reducing immediate disease burden (see above).  However, repeated reliance on AMU contributes to AMR and reduces incentives to invest in more structural approaches to disease prevention, such as infection prevention and control (IPC), biosecurity, and quality herd management practices.  Over time, production systems have become increasingly dependent on AMU to maintain animal and crop health. While this dynamic was recognised overall across countries, some exceptions were also discussed. For example, in the Netherlands, some producers had undergone successful shifts to slower-growing livestock varieties that were less prone to illness and relied less heavily on AMU. Differences between types of producers were also noted: in Canada, for example, stakeholders perceived that larger scale producers could sometimes afford to implement more rigorous practices around IPC, leading to more judicious AMU. | This structure matches the Shifting the Burden archetype because a readily available symptomatic solution (AMU) addresses the immediate problem of disease while delaying or displacing investment in structural solutions that would reduce the underlying need for antimicrobials. As reliance on the symptomatic solution increases, dependency on it is reinforced, and it becomes challenging to escape the system’s behaviour. | Systems theory suggests that this archetype can be disrupted by strengthening the system's capacity to address the underlying causes of disease, thereby reducing reliance on the symptomatic solution.  We therefore reviewed actions proposed by stakeholders to identify actions that emphasised these more ‘structural’ solutions that had been discussed during the modelling process as alternatives that could reduce overreliance on AMU. These included strengthening IPC and biosecurity, improving waste management to limit environmental transmission of AMR, investing in alternative prevention and treatment approaches (e.g., vaccination, phage therapy and gene editing), and supporting livestock health more broadly through feed quality and better production environments. |
| Success to the successful | CLDs showed that antimicrobial-intensive production systems can achieve lower production costs and lower food prices, increasing their competitiveness and market share.  As antimicrobial-intensive systems become more dominant, consumer expectations for inexpensive food are reinforced, further favouring antimicrobial-intensive production and making it more difficult for lower-antimicrobial production systems to remain economically viable.  This dynamic could become entrenched at different scales. In the Netherlands, the profitability of the food production sector was seen as supporting its economic importance and related political influence, enabling push-back to stricter regulations. This created a set of norms around AMU which, while well-regulated and low compared to global averages, were still seen as higher than desirable. In Senegal, challenges in enforcing regulations and the existence of an elicit market for antimicrobials meant that individual producers could seek greater ‘success’ obtained by using antibiotics for growth promotion. | This structure matches the Success to the Successful archetype because one production model accumulates competitive advantages that reinforce its continued success, while alternative production systems become progressively disadvantaged through reduced market opportunities and investment. The reinforcing feedback between market dominance and consumer expectations perpetuates reliance on antimicrobial-intensive production. | Systems theory suggests that this archetype can be disrupted by **levelling the playing field**, reducing the reinforcing advantages of the dominant system while strengthening alternatives.  We therefore reviewed stakeholder recommendations to identify actions that either (1) reduced the advantages for antimicrobial-intensive production systems; or (2) increased the advantages for production systems that were less reliant on antimicrobials.  For (1) we identified actions like enforcing withdrawal period regulation or monitoring antimicrobial residues, measures that create incentives for elevated users of antimicrobials to lower their use.  For (2), we identified actions like providing financial support to enable transitions to species or production systems that are less reliant on antimicrobials, or generating or amplifying consumer demand for products from systems characterised by judicious AMU  through public engagement and labelling schemes.  These measures could be used to level the playing field, attenuating the advantages to be gained from more antimicrobial-intensive production systems. |
